# Developing a Physical Activity Ontology to Support the Interoperability of Physical Activity Data

**DOI:** 10.2196/12776

**Published:** 2019-04-23

**Authors:** Hyeoneui Kim, Jessica Mentzer, Ricky Taira

**Affiliations:** 1 School of Nursing Duke University Durham, NC United States; 2 Department of Radiological Science University of California Los Angeles Los Angeles, CA United States

**Keywords:** exercise, leisure activities, health information interoperability, terminology as topic

## Abstract

**Background:**

Physical activity data provides important information on disease onset, progression, and treatment outcomes. Although analyzing physical activity data in conjunction with other clinical and microbiological data will lead to new insights crucial for improving human health, it has been hampered partly because of the large variations in the way the data are collected and presented.

**Objective:**

The aim of this study was to develop a Physical Activity Ontology (PACO) to support structuring and standardizing heterogeneous descriptions of physical activities.

**Methods:**

We prepared a corpus of 1140 unique sentences collected from various physical activity questionnaires and scales as well as existing standardized terminologies and ontologies. We extracted concepts relevant to physical activity from the corpus using a natural language processing toolkit called Multipurpose Text Processing Tool. The target concepts were formalized into an ontology using Protégé (version 4). Evaluation of PACO was performed to ensure logical and structural consistency as well as adherence to the best practice principles of building an ontology. A use case application of PACO was demonstrated by structuring and standardizing 36 exercise habit statements and then automatically classifying them to a defined class of either sufficiently active or insufficiently active using FaCT++, an ontology reasoner available in Protégé.

**Results:**

PACO was constructed using 268 unique concepts extracted from the questionnaires and assessment scales. PACO contains 225 classes including 9 defined classes, 20 object properties, 1 data property, and 23 instances (excluding 36 exercise statements). The maximum depth of classes is 4, and the maximum number of siblings is 38. The evaluations with ontology auditing tools confirmed that PACO is structurally and logically consistent and satisfies the majority of the best practice rules of ontology authoring. We showed in a small sample of 36 exercise habit statements that we could formally represent them using PACO concepts and object properties. The formal representation was used to infer a patient activity status category of sufficiently active or insufficiently active using the FaCT++ reasoner.

**Conclusions:**

As a first step toward standardizing and structuring heterogeneous descriptions of physical activities for integrative data analyses, PACO was constructed based on the concepts collected from physical activity questionnaires and assessment scales. PACO was evaluated to be structurally consistent and compliant to ontology authoring principles. PACO was also demonstrated to be potentially useful in standardizing heterogeneous physical activity descriptions and classifying them into clinically meaningful categories that reflect adequacy of exercise.

## Introduction

### Challenges in Reusing Physical Activity Data

Undoubtedly, a healthy lifestyle, especially being physically active, is paramount to healthy living. Numerous scientific studies have shown the direct impact of physical activity on disease onset and progress as well as treatment outcomes [1-7]. Although analyzing physical activity data in conjunction with other clinical and microbiological data will lead to new insights crucial for improving human health, its execution is challenging because of the large variation in the way the data are collected and presented.

The first challenge relates to the heterogeneous nature of the acquired data for measuring and assessing physical activity. High-resolution temporal samples of physical activity data captured through personal sensor devices are now becoming increasingly available and feasible for ubiquitous monitoring. Questionnaire-based descriptive measures are also widely used to assess one’s overall exercise habits and factors affecting one’s ability and desire to be physically active. These questionnaire-based measures complement objective measures associated with sensor devices. Each measure poses challenges to reusing the data that it generates. This study concerns improving the reusability of the descriptive measures generated based on questionnaires and assessment scales.

The second challenge relates to the usability of the data. Physical activity data are not free from a common barrier to utilizing text data, which is transforming the data into a computable—that is, structured and standardized—format [8,9]. For example, physical activity data are usually described with nontechnical terms in a lengthy sentence and often buried in narrative notes produced during a clinical encounter. A sample clinical question asked might appear as the following [10]:

Over the past 7 days, how often did you engage in light sport or recreational activities such as bowling, golf with a cart, shuffleboard, fishing from a boat or pier or other similar activities?

Use of lengthy descriptive sentences can help to clearly convey the intention of the question by minimizing the room for misinterpretation. However, it also creates a challenge to systematically analyzing such data in conjunction with other clinical and biological data.

### Limitations of the Existing Standardization Approaches to Representing Physical Activity Data

Common data elements (CDEs) are used in several disciplines for standardizing data collected with assessment scales and questionnaires and have been widely adopted for acquiring self-reported data including physical activity information [11]. The consensus measures for Phenotypes and eXposures (PhenX) Toolkit is a collection of standard measurement protocols that can be used in biomedical research, developed through the cooperative agreement between RTI international and National Institute of Health [12]. PhenX offers a number of standardized scales and questionnaires recommended for collecting physical activity data [12]. Part of the PhenX measures are now included in Logical Observation Identifiers Names and Codes [13]. The CDE repository of the National Library of Medicine allows users to search standardized data elements in the biomedical domain and provides rich metadata on the queried data element, including standardized concept codes [14].

CDEs are an effective method of standardizing questionnaire-based data. However, there are a number of studies involving locally developed questionnaires not covered by the CDE-based standardization efforts. Often, there is a substantial informational overlap within the various questionnaires. For example, there are questions that relate to highly similar topics but belong to different questionnaires and are thus treated as distinctive CDEs. For example, “In the past 7 days, how many days were you physically active for 10 minutes or more?” and “Physical activity 20 minutes per day during week count” both ask for the number of days in a week that a person is physically active although different activity durations are indicated. The former is a question from the Quality of Life in Neurological Disorders questionnaire [15], and the latter is from the National Institute of Neurological Disorders and Stroke questionnaire [16]. Systematically recognizing their similarities will facilitate interoperability of physical activity–related data.

Physical activity is 1 of the 9 social and behavioral health domains that need to be incorporated into electronic health records (EHRs) in a structured format, as recognized by the Office of National Coordinators (ONC) and the Institute of Medicine (IOM—currently the National Academy of Medicine) [17]. Furthermore, ONC and IOM recognized the following 2 salient questions from Exercise Vital Sign [18] as candidate measures to assess physical activity:

1. On average, how many days per week do you engage in moderate to strenuous exercise (such as walking fast, running, jogging, dancing, swimming, biking, or other activities that cause a light or heavy sweat)?
2. On average, how many minutes do you engage in exercise at this level?

These 2 questions certainly provide minimum necessary information on a patient’s overall exercise habit. However, incorporating physical activity as a care regimen or investigating how it affects health outcomes requires a more detailed and diverse representation of one’s physical activity level. In addition, the challenges to reusing the existing physical activity data described for a patient within the EHR remain formidable, including issues related to ambiguity and semantic inconsistency. Furthermore, these data are still largely buried in clinical narrative texts with highly variable forms of expression. Thus, given the increasing clinical awareness of the importance of physical activity assessment, there is a pressing need to explore defining an expanded representation for physical activity data that complements and consolidates existing standardization efforts.

### Gaps in the Existing Ontologies for Physical Activity Data

Many ontologies and standardized terminologies cover aspects of the physical activity domain, but concept coverage remains incomplete. We reviewed existing relevant ontologies and terminology systems to benchmark their structure and to aggregate relevant concepts for our proposed Physical Activity Ontology (PACO). The Semantic Mining of Activity, Social, and Health data (SMASH) ontology contains 74 concept classes that cover the concepts related to social activities and network [19]. SMASH has a well-developed Physical Activity type hierarchy that is divided into Athletic Sports, Exercise, and Occupational Activity. However, its concept coverage on activity types is quite limited, and it does not offer modifier concepts required to describe intensity and amount of physical activity. The Ontology for assessing Physical Activity and Sedentary Behavior (OPA) provides formal expressions for the various domains of concepts relevant to physical activity [20]. OPA focuses on formally representing main top-level concept classes such as *TemporalEntity*, *SpaceEntity*, *Person*, *SocialContext*, etc, and concept properties that link the concept classes. OPA is designed to be used in conjunction with other terminology systems. Therefore, it does not include detailed concepts that belong to the classes. The Ontology of Physical Exercises (OPEs) is a Web Ontology Language (OWL) formatted ontology developed to support consistent representation of exergame data [21]. OPE has comprehensive coverage of concepts important to represent exergame data including game equipment types, health outcomes, engaged musculoskeletal systems, and disease and injuries. OPE also contains some exercise concepts as general categories such as aerobic exercise, isometric exercise, light exercise, etc. Naturally, OPE has many limitations to be considered as an ontology to support representing nongame-based physical activities with sufficient details.

### Study Aim

Consistent and unambiguous representation of physical activity data is essential to draw increased insights that support patient care and health outcome research. There is a need to identify a robust and systematic approach to structuring and standardizing heterogeneous descriptions on one’s physical activity expressed with various measures. The aim of this study was to develop an ontology for physical activity with the concepts important to describe clinically meaningful characteristics of people’s physical activity.

## Methods

### Data Sources

We collected 1140 unique questions and sentences on physical activity from 92 questionnaires and forms such as Healthy Living Questionnaire [22], Rapid Assessment of Physical Activity [23], Two Question Physical Activity Assessment [24], etc. The full list of questions and sentences analyzed in this study is provided in Multimedia Appendix 1.

### Multipurpose Text Processing Tool

Exploration of the concepts and terms referenced within the 1140 unique questions were facilitated using a natural language processing (NLP) tool called Multipurpose Text Processing Tool (MUTT) developed by the Medical Imaging Informatics Group at the University of California Los Angeles. This NLP environment is designed to allow developers to both define the ontologic elements and structure of the target domain and link corresponding NLP lexico-syntactic-semantic patterns to identify them in free text. This approach is similar to recent ontology-driven NLP applications such as is employed in the OpenDMAP project [25]. The NLP pattern acquisition aspect is data driven, similar in this respect to the knowledge discovery methods reported in another study [26]. Details of the core NLP system can be found in other studies [27-29].

**Figure 1:**
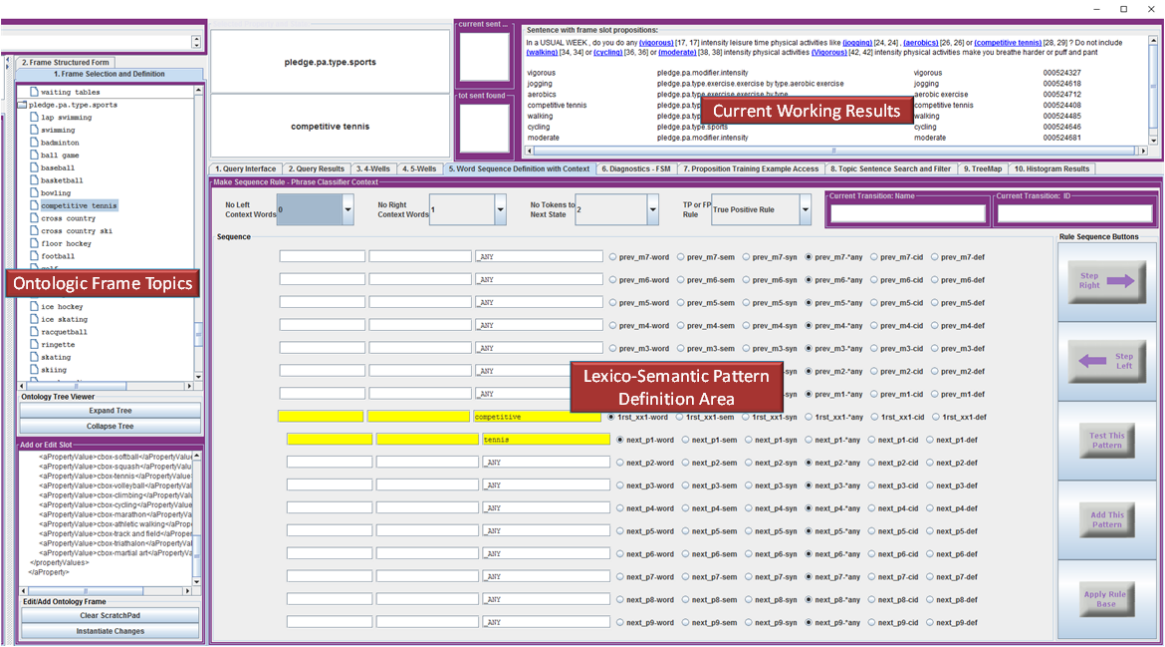
Screenshot of the multipurpose text processing tool user-interface environment. The Ontologic Frame Topics box displays an ontology specification with XML and tree representations; Users specify lexico-syntactic-semantic patterns of the text corresponding to ontologic property instance in Lexico-Semantic Pattern Definition Area; The Current Working Results box provides immediate user feedback to specified patterns on training sentence. Not shown are user’s screens to view training sentences, training status, and automated pattern discovery results.

The main MUTT module interface is shown in Figure 1. The first step is to define a topic class (eg, PACO) using a free text XML editor. This high-level class allows various NLP extraction modules related to this class to be activated. The next step involves defining the hierarchy of concepts under the topic class. Once a user has defined an ontological class definition (eg, exercise.equipment) and its possible instances (eg, treadmill, rowing machine, or elliptical), the next step involves defining NLP extraction patterns to identify such instances in free text. This step allows the system to precompile a knowledge source that serves as a mapping between ontological instances and all their associated lexical variant patterns.

Practically, users can instantiate extraction patterns best by viewing training examples (ie, a particular question) and using the Figure 1 interface to define concept detection patterns. With this approach, the following steps are performed: (1) user selects a training sentence; (2) the system tokenizes the sentence and displays all ontologic concepts it currently can extract (see the Current Working Results area of Figure 1); (3) the user examines the results and can decide if there are any ontologic terms missing from the extracted sentence results; (4) as part of the results for the selected sentence, the system presenting the user with a scrollable table listing 1 word token per line with column fields corresponding to exclusively selectable matching attributes of the token including its exact surface string, semantic class, part-of-speech class, wildcard and the user definable and selectable attributes of predefined morphological features, and token-level regular expression; and (5) the user can define an extraction pattern across multiple tokens (ie, multiple word phrases) in this manner, with each token within the sequence specified separately. The interface also allows users to specify left and right context tokens to partially address issues of semantic ambiguity within the context of the question. Patterns can be associated with either a true positive or a false positive match. Many such patterns can be associated with a single ontologic concept.

**Table 1:** Sequence of training and testing steps for term extraction and the number of sentences used for training and testing.

Step	Text data	Annotator	Task
1	Set 1 (n^a^=100)	Human	Developing baseline semantic models
2	Set 2 (n=400)	Human	Round 1 training
3	Set 3 (n=100)	MUTT^b^	Round 1 testing
4	Test number 1 results	Human	Reviewing and analyzing the round 1 testing results
5	Set 4 (n=300)	Human	Round 2 training
6	Set 5 (n=240)	MUTT	Round 2 testing
7	Test number 2 results	Human	Reviewing and analyzing the round 2 testing results

^a^n=number of sentences included in the annotation set.

^b^MUTT: multipurpose text processing tool.

### Harvesting Terms

We first structured 100 questions by annotating key concepts based on a preliminary concept model from a previous effort [30]. This preliminary model comprises 3 concept classes including activity type, modifiers, and facilitating and inhibiting factors. The model also includes semantic relations among these classes. Through this initial analysis, we further specified and expanded the base concept classes in the preliminary concept model by creating multiple child classes. Activity types were divided into *exercise, daily activity (including household chores)*, and *leisure/recreational activities.* Modifiers were further detailed into *amount, frequency,* and *intensity*. We also identified additional concept classes important to capture the concepts describing people’s activity level during this initial annotation. For example, *exercise location*, *exercise equipment*, and *fitness program/classes* were added. Note that our goal of modeling at this point was to be as comprehensive as possible.

The baseline model was populated as the initial semantic frame model in MUTT. The remaining 1040 sentences were annotated using MUTT. Text annotation and the semantic model augmentation process were performed iteratively by 2 annotators as illustrated in Table 1.

### Building an Ontology

The ontologic frame definitions manually specified using the MUTT interface were converted from XML to OWL and subsequently imported as a baseline ontology into Protégé, an ontology authoring tool developed by Stanford Center for Biomedical Informatics Research at the University [31]. We also searched the National Center for Biomedical Ontology BioPortal [32] for any ontology relevant to physical activity to incorporate additional concepts and relations. BioPortal is a Web service that allows users to upload, search, and access biomedical ontologies, developed and maintained at the Stanford University. We reviewed ontologies retrieved with the search term *exercise* and *physical activity*, which yielded 48 ontologies or standardized terminology systems including Systematized Nomenclature of Medicine-Clinical Terms [33], Medical Subject Headings [34], the National Cancer Institute Thesaurus [35], and the Read Clinical Terminology Version 2 [36]. Most of these ontologies have a small substructure (a branch or a single class) relevant to physical activity. We found that majority of the concepts found in these ontologies were already included in our baseline ontology. Through this activity, we added 1 new concept, *high intensity interval training*. Cross-referencing previous ontologies in addition provided an opportunity to ensure that our concepts were appropriately phrased and placed at an appropriate level in the hierarchy.

Naming conventions of class labels used the singular form of nouns and verbs with the first letter of a word being capitalized. An underscore is inserted between words for a multiword label (eg, *Ice* _ *hockey* and *Circuit_training*). Sport or exercise names are included in a noun form. We adopted a gerund form if a concept has only a verb form. Instances (Individuals in Protégé) are labeled in all lowercase and likewise, an underscore is inserted between words for a multiword instance label (eg, *high_impact* and *make_you_puff_and_pant*). Property names followed a camel case style (eg, *hasIntensity* and *hasActivityEffect*).

Overall, 2 general activity classes were defined at a top level that differentiated daily (*Daily_living_activity)* versus leisure (*Exercise_leisure_activity)* activities. These classes were then further divided into a number of subclasses. There are multiple ways to categorize the activity names under *Exercise_leisure_activity*. For example, dancing is a physically active leisure activity and at the same time can be considered a comprehensive exercise that helps with endurance, flexibility, balance, and bone and muscle strength. *Cross_country_ski* can be classified as a winter outdoor sport in addition to the categories defined by its exercise effects.

To efficiently handle the complexities in classifying activity types, we adopted an asserted hierarchy and a defined hierarchy. We asserted the hierarchy of *Exercise_leisure_activity* using apparent and more generic subsumptive relations. For example, the *Ballgame* class contains different ball games such as *Soccer, Baseball, Tennis*, etc. Similarly, the *Running* class contains various exercise and/or sports characterized by running such as *Jogging*, *Treadmill_running*, *Sprinting*, *Marathon*, etc. Additional activity type classes were created as a defined hierarchy to incorporate a few common ways of categorizing exercise and leisure activities such as by exercise effects, by indoor or outdoor activity, winter activity, and water activity. These multiple views of organizing the ontology were implemented using a multiple-inheritance structure. We defined each named activity under the *Exercise_leisure_activity* class with the 3 properties of *hasActivityEffect*, *hasActivityLocation*, and *hasActivityRequiredCondition*. We then generated an inferred hierarchy where these named activities are classified under the defined classes. This inferred hierarchy was specified using FaCT++, a Web Ontology Language Description Logic (OWL-DL) reasoner available in Protégé [37]. FaCT++ is an open-source software developed by Dmitry Tsarkov and Ian Horrocks at the University of Manchester [37]

### Ontology Evaluation

We checked the logical and structural quality of PACO first using the Ontology Debugger plugin available in Protégé [38]. We also tested PACO with the Ontology Pitfall Scanner! (OOPS!) tool [39] to ensure its compliance to the ontology authoring principles in addition to the structural quality. OOPS! is a Web-based tool from the Ontology Engineering Group of the Technical University of Madrid that examines an ontology against 33 common pitfalls as compared with state-of-the-art principles for ontology construction. These pitfalls cover not only logical and structural issues but also usability and documentation concerns [40].

**Figure 2:**
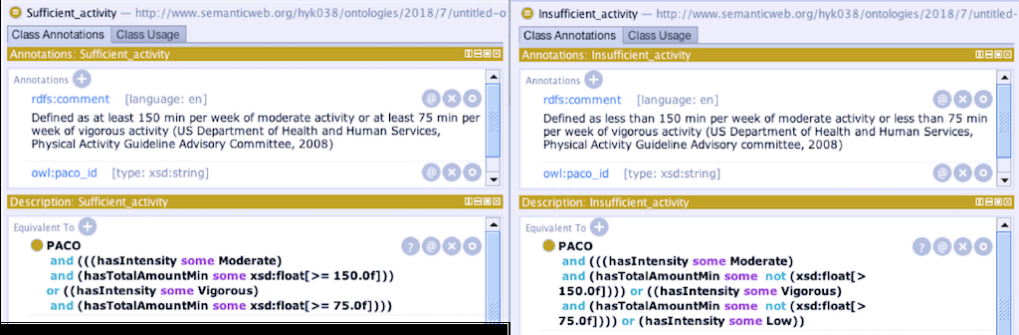
Defining exercise level classes.

As an additional quality assurance effort, we tested the system’s ability to identify ontologic concepts from various free-text physical activity descriptions. Clinically, 2 important outcome nodes were added for this step that represent adequacy of exercise, that is, *Sufficient_exercise* and *Insufficient_exercise*. These classes were defined using the following 2 properties: *hasIntensity* that captures an intensity level and *hasTotalAmountInMin* that captures total weekly exercise amount measured in minutes (see Figure 2). The definitions of the 2 exercise levels were developed based on the physical activity guideline provided by the US Department of Health and Human Service in 2008 [41].

We collected 36 descriptions on usual exercise habits from Web consumer-oriented behavioral health articles (eg, World Health Organization and American Heart Association) and from a convenience sample of 30 people (ie, friend, family, and colleagues of the authors). We formally defined these 36 statements using *hasIntensity* and *hasTotalAmountInMin*, and then added them to PACO as an instance (ie, individual in Protégé) of a specific exercise type. We evaluated the concept coverage of the *Exercise_leisure_activity* class by identifying an exercise type that an instance belongs to and by populating the intensity property with an intensity concept from the Intensity class. We classified the 36 exercise statements into 1 of the 2 exercise level classes using FaCT++.

## Results

### Natural Language Processing Term Extraction Performance

Upon completion of processing the corpus of 1140 unique sentences on physical activity, 268 unique terms and concepts were structured into 33 semantic frames. Table 2 presents the performance of MUTT on identifying relevant physical activity terms and concepts obtained from the 2 rounds of evaluation. We manually reviewed the annotation results in MUTT. The result window of MUTT (see the Current Working Results area of Figure 1) allows users to review the terms and phrases captured by MUTT within the sentence that they belong to. Therefore, users can easily determine whether the recognized terms are relevant or any relevant terms are missed. The first round of evaluation was done with a collection of 100 unique sentences. MUTT reached an *F* score of 0.895 with this evaluation. After an additional training round, MUTT’s performance was improved when evaluated with a second set of 240 unique sentences. With the *F* score of 0.950, the terms and concepts extracted using MUTT were deemed sufficiently comprehensive. No incorrect (ie, false positive) annotation was observed in either test set.

### Physical Activity Ontology

The 268 unique terms and concepts identified with MUTT and 1 additional concept identified from the existing ontologies were structured into the PACO. PACO currently contains a total of 225 concept classes including 1 root class, which we label as PACO, and 9 defined classes. PACO contains 20 object properties (including 10 inverse properties) and 1 data property called *hasTotalAmountMin*. The main concept hierarchy is formed with 5 branches of *Activity*, *Exercise_effect*, *Exercise _equipment*, *Exercise_program*, and *Modifier.* Most of the prepared concepts were placed under the *Activity* and *Modifiers* branches, which are structured into multiple layers of classes. *Activity* is the largest branch that includes 2 primitive classes and 7 defined classes, which span to 4 subclass levels. The 2 primitive classes are *Daily_living_activity* that contains various household chores and home maintenance activities and *Exercise_leisure_activity* that includes various exercise, sports, and other hobbies involving physical activity such as dancing, fishing, and camping. Overall, 4 of the 7 defined classes are formed by exercise effects and include *Balance_exercise*, *Endurance_exercise*, *Flexibility_exercise*, and *Strength* _*exercise*. The remaining 3 classes are *Outdoor_leisure_activity*, *Water_sport*, and *Winter_sport*. These 7 defined classes support additional views that are commonly used to classify these physical activities.

**Figure 3:**
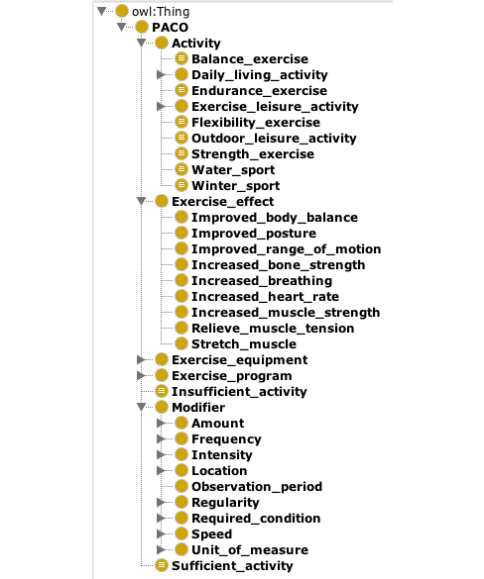
Physical activity ontology high-level hierarchy.

The largest concept class is *Exercise_leisure_activity* under *Activity*, which contains 38 subclasses. The *Exercise_equipment* and *Exercise_program* branches are quite small and incomplete, and each contains only 7 and 3 subclasses, respectively. A number of expressions that represent various intensity levels such as “until sweat a lot and breathe hard” and “makes heart rate increase a bit” were instantiated under the 3 intensity-level classes of *Low, Moderate*, and *Vigorous*. Figure 3 shows the high-level hierarchy (asserted) of PACO. The PACO structural summarization metrics are presented in Table 3. PACO has been included in BioPortal [42].

**Table 2:** Multipurpose text processing tool annotation performance, and the number of sentences, terms and concepts used in the evaluation.

Test number	Sentences, n	Target terms and concepts, n	Recall	Precision	*F* score	Example terms or expressions missed
1	100	292	0.857	1.000	0.895	hike uphill, walk uphill, ride a bike, walk fast, exercise (generic), how many total hours, etc
2	240	443	0.940	1.000	0.950	push-ups, weightlifting, brisk walking, washing clothes by hand, calisthenics, squash, etc

**Table 3:** The number of axioms and entities included in the Physical Activity Ontology.

Axioms and Entities in the Ontology	n
Class	225
Defined class	9
Maximum depth of classes	5
Minimum number of siblings	2
Maximum number of siblings	38
Logical axiom	587
Declaration axiom	297
Axiom for subsumption relations (subclass of)	397
Object property (excluding inverse property)	10
Data property	1
Instance (excluding 36 exercise statements)	23

### Physical Activity Ontology Evaluation

PACO provided all activity types and intensity concepts that were required to represent the 36 exercise statements. The 36 exercise statements were all correctly classified into either the sufficient or insufficient level of exercise classification using FaCT++.

The results of the Protégé Ontology Debugger program indicated that PACO has well-defined concept classes, and all individuals are consistently instantiated under the relevant classes. However, OOPS! [39,40] caught a few issues in PACO. Absence of inverse object properties and absence of annotation of each class were identified as a minor problem. Interestingly, OOPS! recognized 3 pairs of potentially equivalent concepts—2 temporal concepts *Minute* and *Hour, Hockey* and *Field_hockey*, and *Hockey* and *Ice_hockey.* Not explicitly declaring their equivalence was identified as an important problem. OOPS! does not recognize the license information included as a PACO metadata, and absence of ontology license information was considered another important problem. We modified PACO to address these problems when possible. We added 10 inverse object properties that correspond to the 10 object properties. We annotated every class with internal concept identifier. We renamed Hockey to *Hockey_game* because ice hockey and field hockey are often called simply hockey in real-world communications.

## Discussion

### Principal Findings

We developed PACO as a conceptual foundation for systematically structuring and standardizing physical activity descriptions. PACO includes specific activity types and modifiers that are frequently used to further specify different properties of an activity. PACO was evaluated using the Ontology Debugger program of Protégé and the OOPS! program to ensure structural consistency and compliance to well-accepted ontology building principles.

Physical activities are often described with nontechnical terms and can be expressed in various forms that may include precise numeric measures (eg, walks 3 miles after dinner 1-2 times per week) to general colloquial descriptors (eg, occasionally go for a long walk after dinner). Existing biomedical terminology systems offer limited coverage for physical activity names and general descriptors. Instead of proposing to include more concepts and terms to the existing terminologies, we developed PACO, an ontology for a specialized scope of physical activities to reap the full benefit of an ontological approach to concept representation. For example, a collection of individual terms mapped to a standardized terminology does not capture the complete meaning as the semantic relations between the terms are not explicitly represented. PACO supports expressing complex concepts by linking physical activity concepts and modifier concepts using object properties based on the predefined semantics described in the ontology. In addition, activity names and types are classified from multiple perspectives in PACO using various classification criteria. These can be supported by concept post coordination with a compositional terminology that supports multiple inheritance. However, it is a nontrivial task to fully address diverse and complicated representational needs of physical activity descriptions for all potential queries that may originate from a broad scope of biomedical domains and applications.

PACO is one of the first ontologies that is dedicated to representing the concepts related to physical activity. PACO incorporates not only conceptual models but also individual concepts important to describe one’s physical activity level such as activity types, intensity, and amount. PACO has a relatively simple asserted hierarchy where new concepts are easily added. To accommodate multiple ways of classifying physical activities, several defined classes were added and a multiple inheritance structure (ie, inferred hierarchy) was generated using a publicly available OWL-DL reasoner.

### Limitations and Future Enhancement of Physical Activity Ontology

PACO contains concepts mainly derived from various assessment scales and questionnaires on physical activity. Therefore, it may have missed the concepts used in other types of physical activity descriptions found in various text sources such as patient exercise diary, clinical notes, and research articles. The *Activity* branch contains a number of specific types of physical activities including daily living activities and exercise/leisure activities that are frequently used to describe physical activity types in assessment scales and questionnaires. Although the *Activity* branch has the largest number of nodes and the deepest structure in PACO, it by no means contains the exhaustive set of activity type concepts. The *Activity* branch will continuously expand as more related sources of text are analyzed and incorporated. The multiple inheritance structure will also adaptively evolve as additional ways of classifying activities are identified.

Many physical activity questions employ phrases that describe physical responses to exercise such as “...[exercise] until you breathe harder than normal” or “...[exercise] makes you puff or pant,” in addition to the general adjectives such as mild, moderate, strenuous, vigorous, etc. For example, 1 question of the Exercise Vital Sign uses an intensity descriptor of “... causes a light or heavy sweat,” which indicates moderate or vigorous exercise. Although these “raw” expressions sound somewhat subjective, incorporating them into PACO was deemed important as exercise intensity can be a subjective experience influenced by people’s age, overall health status, and fitness. These “raw” expressions were included in PACO as an instance of the intensity concept class Mild, Moderate, and Vigorous.

Intensity of exercise can also be captured by the type of activity itself. Many exercise guidelines and activity questionnaires provide specific types of activities as an example of indicating different exercise intensity levels. For example, brisk walking, water aerobics, and yoga are considered moderate activities, whereas jogging, aerobic dancing, and various competitive martial arts are considered vigorous activities [43]. In this version of PACO, named activities are not defined with an intensity level. However, we plan to attach the intensity property to the named activities in a revised PACO if it is deemed useful.

As an example-of-use demonstration, we classified 36 exercise habit statements into 2 clinically relevant exercise levels, that is, sufficient and insufficient, which are defined crudely based on the exercise amount and intensity. This is to show that once various exercise habit statements are formally represented using the concepts and properties in PACO, the formal representation can further be used to logically infer essential information (ie, whether the person is getting an adequate level of exercise or not). In reality, however, determining the adequacy of exercise level requires considering a person’s individual characteristics such as demographics, body measures, health status, and overall physical fitness, in addition to the exercise description itself. A real-world application of algorithmically determining adequacy of one’s exercise level will require incorporating objective measures such as metabolic equivalent of task-minutes (MET-minutes) [44] and heart-rate–based intensity measures (eg, 50%-70% increase of your maximum heart rate for moderate intensity) [45,46]. A future enhancement of PACO can consider including MET values as a property of specific named activities. In addition, exercise-induced heart-rate changes can be incorporated into the definitions of the exercise-level classes.

These limitations and the areas for future enhancements, however, do not detract from the motivating goal of PACO to contribute toward a precision medicine practice by facilitating the integration of heterogeneous data on physical activity generated by various sources. For example, PACO can provide standardized representations for a person’s self-reported subjective descriptions on intensity and adequacy of physical activities. Moderate-intensity activities are considered to have the energy expenditure of 3 to 6 METs [44,47,48]. Walking 4.5 miles per hour falls in the moderate-intensity activity category, but to some people, this level of activity may be perceived as quite a high-intensity activity. Comparing these descriptions with the objective data collected from a person’s mobile sensor device (ie, activity tracker) may lead to refined assessments and recommendations for improving a patient’s physical activity level toward sufficiency [49].

### Conclusions

Physical activity data are an important aspect to understanding general health, disease progress, and treatment outcome. The wide variety of ways of representing one’s physical activity data has become a challenge with regard to analyzing them in conjunction with other clinical and biological data. As a first step toward standardizing and structuring heterogeneous descriptions on physical activity for integrative data analyses, we developed PACO with the concepts collected from physical activity scales and questionnaires. PACO was proven to be structurally consistent and cohesive and also demonstrated to be potentially useful in standardizing heterogeneous physical activity descriptions and classifying them into clinically meaningful categories that reflect adequacy of exercise. PACO will be continuously augmented to expand its concept coverage and semantic properties to support consistent documentation, standardization, and harmonization of physical activity data as described in various textual forms.

## Appendix

Multimedia Appendix 1 Questions and sentences analyzed in this study.

## Abbreviations

CDE: common data elements
DL: Description Logic
EHR: electronic health record
IOM: Institute of Medicine
MET: metabolic equivalent of task
MUTT: Multipurpose Text Processing Tool
NLP: natural language processing
ONC: Office of National Coordinators
OOPS: Ontology Pitfall Scanner
OPA: Ontology for Assessing Physical Activity and Sedentary Behavior
OPE: Ontology of Physical Exercise
OWL: Web Ontology Language
PACO: Physical Activity Ontology
PhenX: Phenotypes and eXposures
SMASH: Semantic Mining of Activity, Social, and Health Data
